# Proteomic Analysis of Nucleus Pulposus Cell-derived Extracellular Matrix Niche and Its Effect on Phenotypic Alteration of Dermal Fibroblasts

**DOI:** 10.1038/s41598-018-19931-9

**Published:** 2018-01-24

**Authors:** Minting Yuan, Pei-Jing Pai, Xiaofen Liu, Henry Lam, Barbara P. Chan

**Affiliations:** 1 Tissue Engineering Lab, Department of Mechanical Engineering, The University of Hong Kong, Pokfulam Road, HKSAR, China; 2 Department of Chemical and Biomolecular Engineering, The Hong Kong University of Science and Technology, Clear Water Bay, HKSAR, China; 3 Division of Biomedical Engineering, The Hong Kong University of Science and Technology, Clear Water Bay, HKSAR, China

## Abstract

Reconstituting biomimetic matrix niche *in vitro* and culturing cells at the cell niche interface is necessary to understand the effect and function of the specific matrix niche. Here we attempted to reconstitute a biomimetic extracellular matrix (ECM) niche by culturing nucleus pulposus cells (NPCs) in a collagen microsphere system previously established and allowing them to remodel the template matrix. The reconstituted NPC-derived complex ECM was obtained after decellularization and the composition of such niche was evaluated by proteomic analysis. Results showed that a complex acellular matrix niche consisting of ECM proteins and cytoskeletal proteins by comparing with the template collagen matrix starting material. In order to study the significance of the NPC-derived matrix niche, dermal fibroblasts were repopulated in such niche and the phenotypes of these cells were changed, gene expression of collagen type II and CA12 increased significantly. A biomimetic NPC-derived cell niche consisting of complex ECM can be reconstituted *in vitro*, and repopulating such matrix niche with fibroblasts resulted in changes in phenotypic markers. This work reports a 3D *in vitro* model to study cell niche factors, contributing to future understanding of cellular interactions at the cell-niche interface and rationalized scaffold design for tissue engineering.

## Introduction

Matrix niche, the local microenvironment including the extracellular matrix (ECM) is among the most important factors for affecting cellular phenotypes and activities, especially for cells exhibiting plasticity such as stem cells^1,2^ and dermal fibroblasts (DFs)^3,4^. Reconstituting matrix niche in a physiologically relevant microenvironment, which cells normally reside, in 3D configuration, will enable us to understand the effect and function of cell matrix niche^5,6^. A biomimetic niche requires the integration of physical and biochemical cues. Acellular ECM derived from native tissues via decellularization provides the structural support and serves as a reservoir of growth factors and cytokines. These decellularized native tissues have been investigated as nature-simulating scaffolds for tissue engineering in blood vessels, nerves, ligaments, heart valves and tendons^7–10^.

Degeneration disc disease (DDD), which is associated with low back pain, has an enormous socioeconomic impact on people’s lives^11^. In comparison to traditional clinical approaches, new treatment modalities, such as tissue engineering and regenerative medicine (which aim to restore disc function and retain mobility, rather than to simply relieve the symptoms or source of pain), have been developed in recent years. Being able to reconstitute the biomimetic microenvironment of the disc represents one approach for developing scaffolds; these are then exposed to cells that exhibit plasticity, such as stem cells or fibroblasts, for tissue engineering purposes^12^. For example, nucleus pulposus (NP)^13,14^, annulus fibrosus (AF)^15^ and whole intervertebral disc (IVD)^16,17^ have been decellularized to create biomimetic scaffolds for potential applications in disc tissue engineering. Our group has developed a collagen microencapsulation technology whereby cells are entrapped in a nanofibrous collagen meshwork^18^, which serves as a template for ECM remodelling. We have previously demonstrated that when rabbit NPCs were cultured in the collagen meshwork, they maintained their normal phenotype^19^. Moreover, the collagen meshwork template was remodelled by the NPCs in a 3D configuration^12^.

DFs are a population of cells of mesenchymal origin in the skin. They have recently gained attention as a promising source of cells for cell therapy and tissue engineering owing to their easy accessibility and robust plasticity^20–23^. Specifically, DFs can be de-differentiated into induced pluripotent stem cells (iPSCs)^24^, and under specific culture conditions, they can be transdifferentiated into cells of other lineages, such as osteocytes^4^, hepatocytes^25^, macrophages^26^, endothelial cells^27^, and chondrocytes^3^, the latter sharing many common features with NPCs^28^.

In this project, we endeavoured to reconstitute a biomimetic NP matrix microenvironment derived from primary nucleus pulposus cells (NPCs) and analysed the composition of the NPC-derived ECM niche and investigated the effect of such niche on cultured human DFs. Our approach was to culture NPCs within collagen microspheres using methods to maintain their normal phenotype, so that they would remodel the template collagen meshwork with an NPC-derived ECM microenvironment. Upon removal of the original NPCs via decellularization, the composition of the reconstituted NPC-derived complex matrix was evaluated in detail by proteomic analysis. In order to study the effect of NPC-derived matrix in affecting cellular phenotype, human DFs were then seeded onto the NPC-derived matrix, after which their viability and phenotypic characteristics at the gene and protein level were assessed. We believe that having a systematic understanding of the molecular composition of the cell-derived matrix and optimization of a biomimetic microenvironment is crucial for rationalized scaffold design for future tissue engineering.

## Results

### Characterization of NPC-derived matrix before and after decellularization

**Figure 1:**
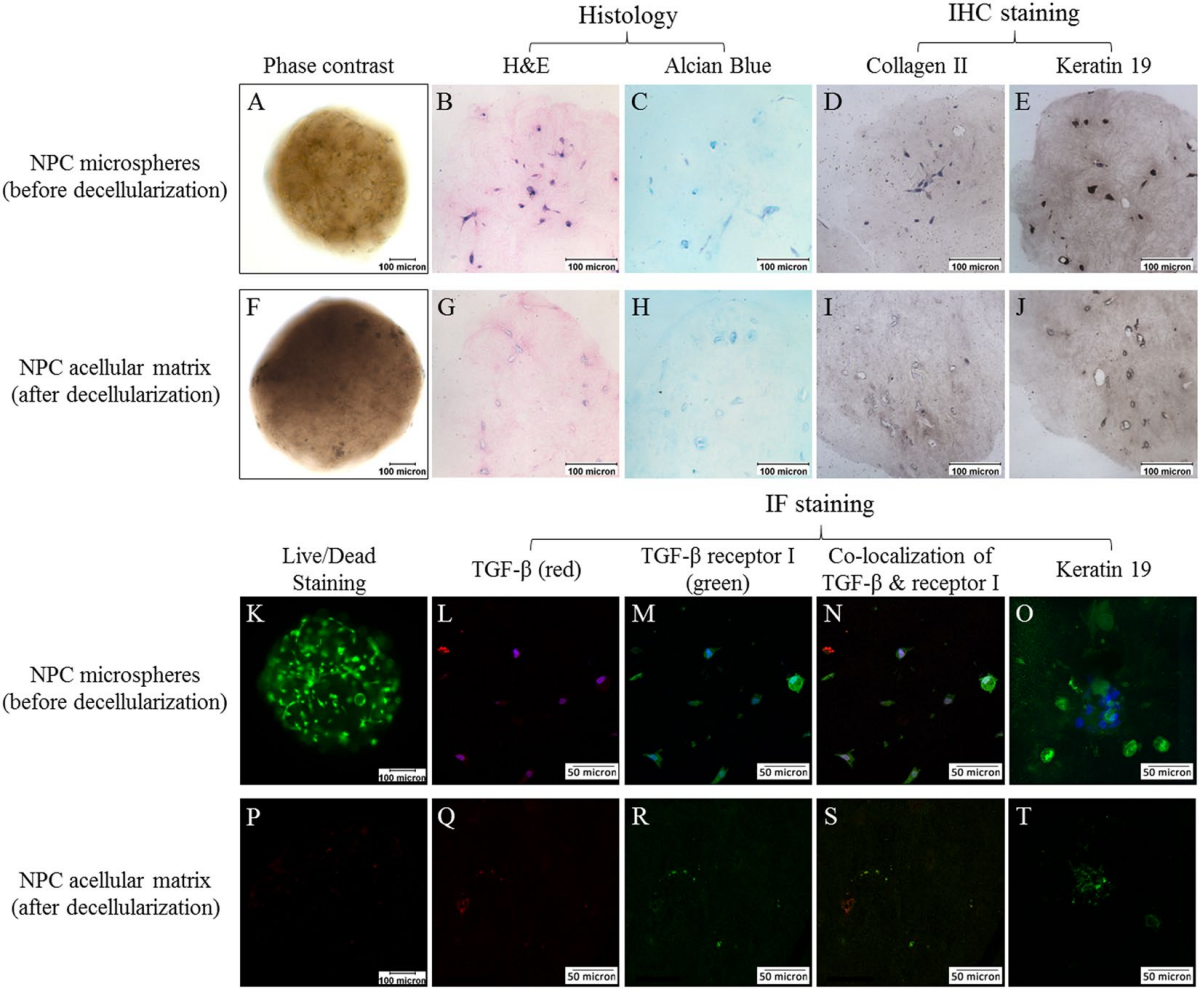
Characteristics of the NPC microspheres before and after decellularization. (**A**–**E** and **K**–**O**) NPC microspheres before decellularization; (**F**–**J** and **P**–**T**) NPC microspheres after decellularization; (**A** and **F**) phase contrast images; (**B** and **G**) hematoxylin and eosin (**H** and **E**) staining; (**C** and **H**) alcian blue staining; (**D** and **I**) immunohistochemical staining of collagen type II; (**E** and **J**) immunohistchemical staining of keratin 19; (**K** and **P**) live/dead staining (green: live cells; red: dead cells); (**L**–**O** and **Q**–**T**) immunofluorescent staining (blue: DAPI); (**L** and **Q**) immunofluorescent staining of TGF-β (red); (**M** and **R**) immunofluorescent staining of TGF-β receptor I (green); (**N** and **S**) co-localization of TGF-β and TGF-β receptor I (white with DAPI; orange without DAPI); (**O** and **T**) immunofluorescent staining of keratin 19 (green). (Scale bars: 50 microns in immunofluorescent staining, 100 microns in the others).

As shown in Fig. 1, ECM components such as GAGs and collagen type II, and the cytoskeletal protein keratin 19, were retained after decellularization. In addition to these ECM and cytoskeletal components, TGF-β (a soluble growth factor) and its membrane-bound receptor TGF-β receptor I, were partially retained after decellularization and found co-localized.

Mass spectrometry analysis showed that before decellularization, there were 220 proteins localized within NPC-microspheres, and that after decellularization more than half of these proteins (i.e., 133 ± 13) were still maintained (Supplementary Table S1).

**Figure 2:**
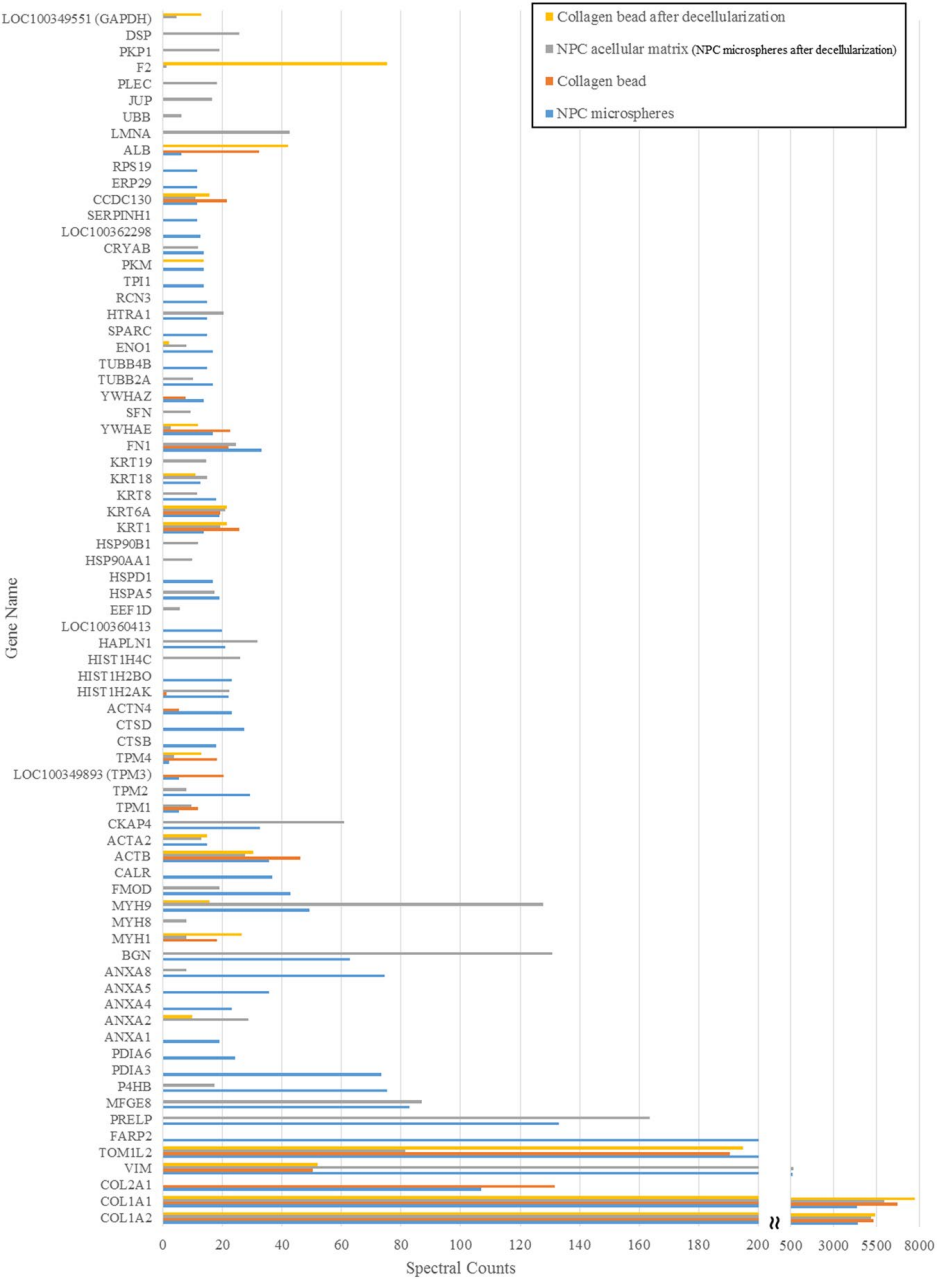
Proteins with spectral counts above 10.

**Figure 3:**
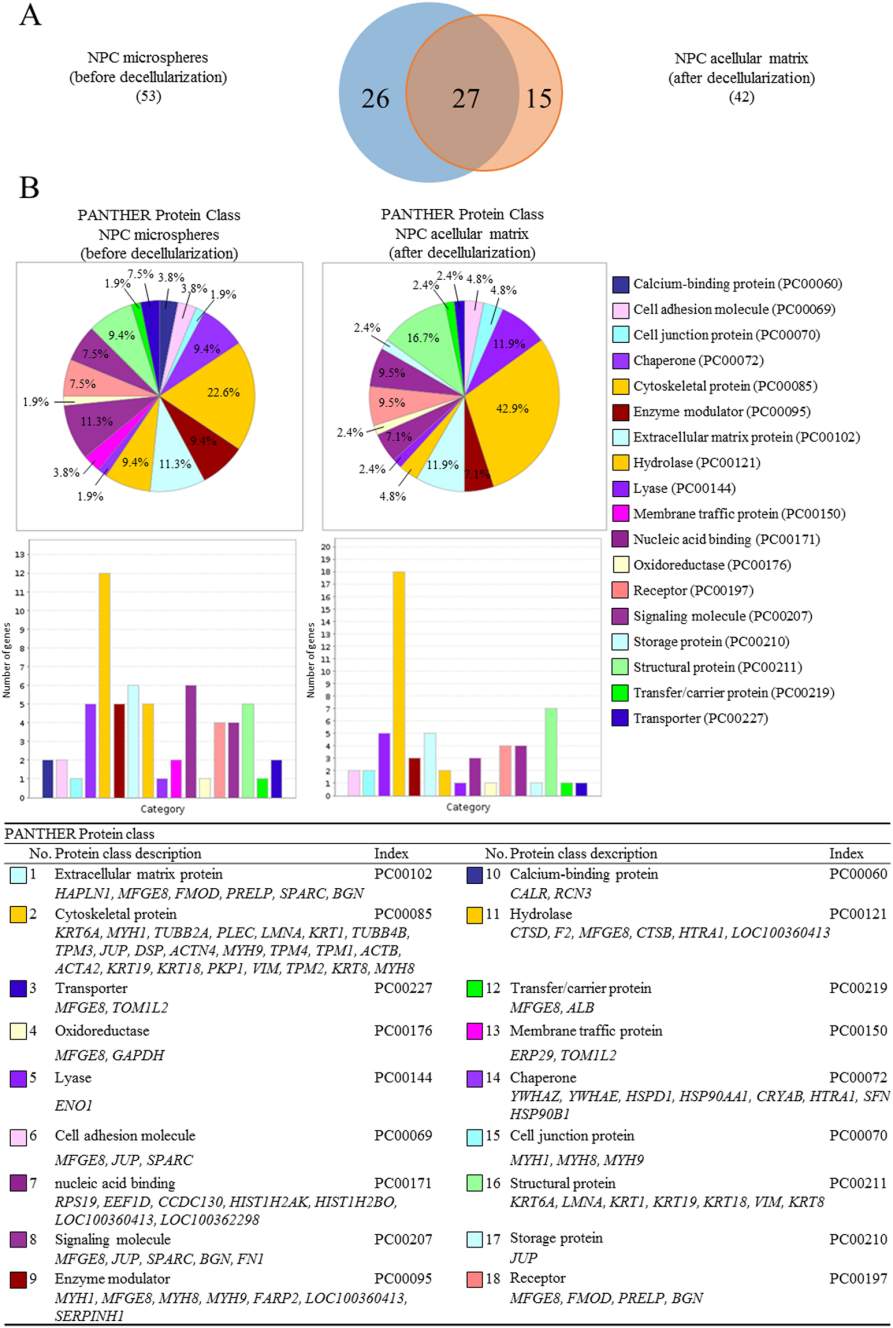
Proteins identified before and after decellularization. (**A**) The number of identified proteins; (**B**) PANTHER protein class.

The spectral counts of each identified protein indicate the relative abundance of the protein under different experimental conditions^29^. Figures 2 and 3, and Supplementary Table S2 showed the identified proteins (along with their PANTHER protein class), which had a spectral count >10, interpreting as relative abundant proteins. Even though the starting material of the microspheres was only collagen type I from commercial sources, according to the mass spectrometry results, other proteins such as serum albumin, at much lower abundance, were identified (Supplementary Table S3). These proteins might come from impurities in the collagen starting material or in the medium supplements used during culture. Therefore, protein IDs with the spectral counts of negative control (collagen beads) two times greater than those in the experimental groups were regarded as being impurities or contaminants (those underlined in Supplementary Table S3) and hence excluded for composition analysis. After excluding the impurities, 53 and 42 proteins were identified before and after decellularization, respectively. Among them, 27 proteins were the same and hence regarded as those retained after decellularization (Fig. 3A). Protein class analysis using PANTHER indicated that differentially expressed candidates in the NPC-collagen microspheres before decellularization were mainly cytoskeletal proteins (22.6%), ECM proteins (11.3%) and nucleic acid binding (11.3%) proteins; whereas after decellularization, cytoskeletal proteins (42.9%), structural proteins (16.7%), ECM proteins (11.9%) and chaperone proteins (11.9%) were most abundant (Fig. 3B).

**Figure 4:**
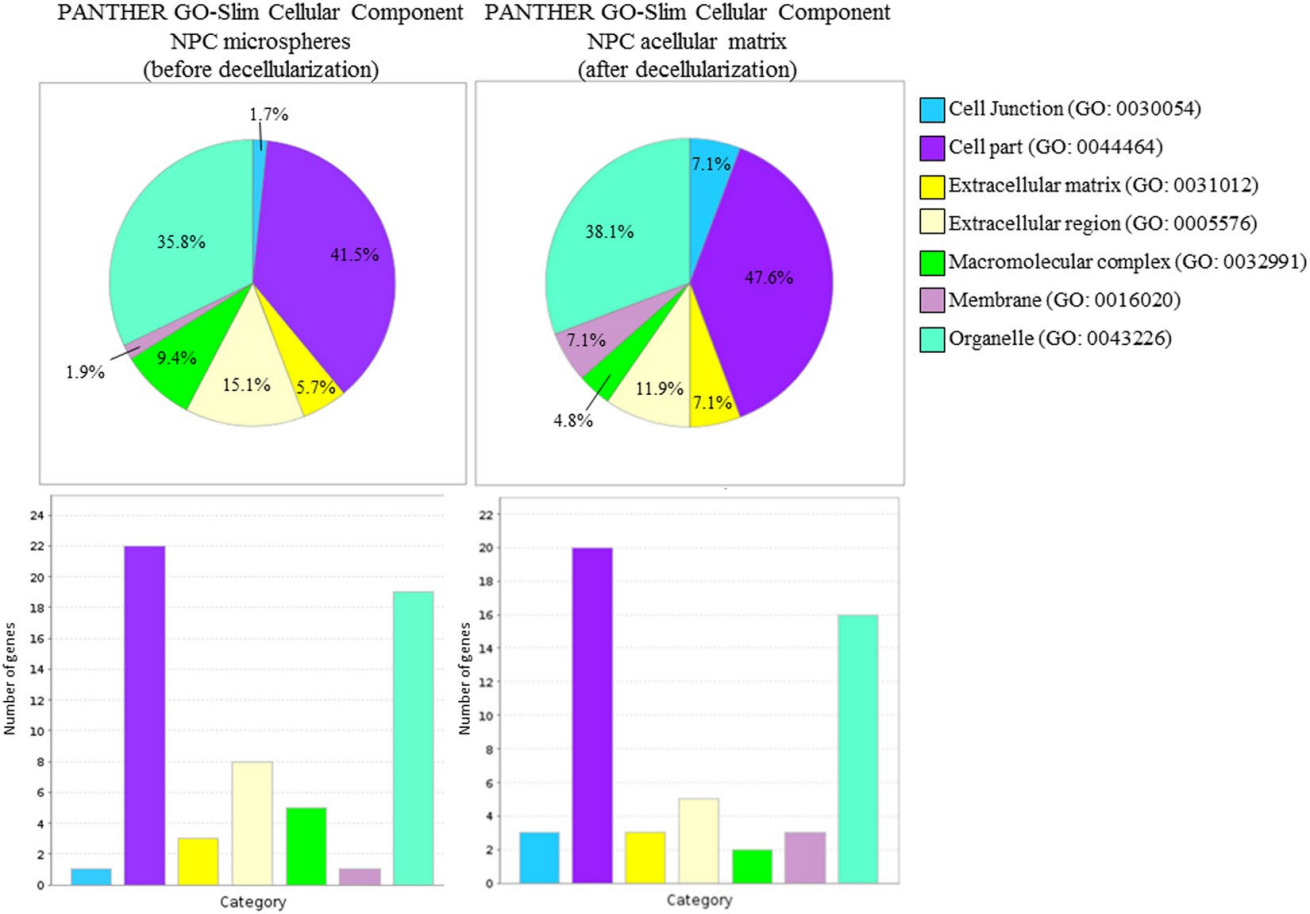
PANTHER GO-slim cellular component of NPC microspheres before and after decellularization.

**Figure 5:**
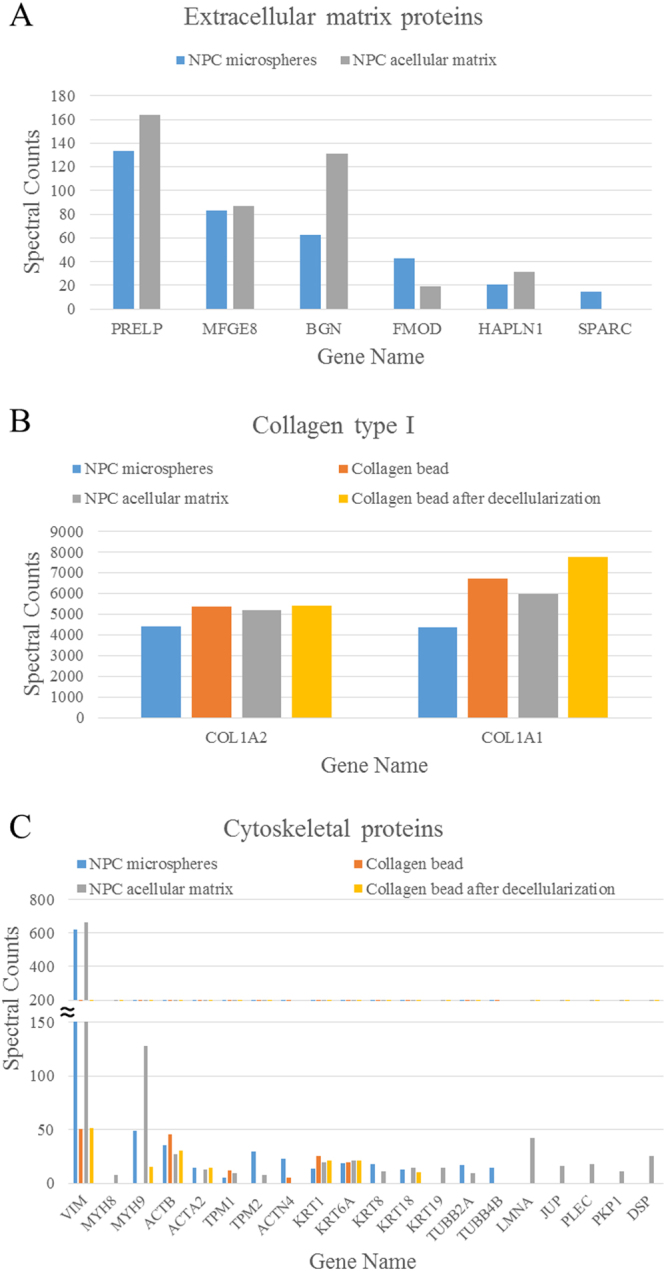
Spectral counts of collagen type I, extracellular matrix proteins and cytoskeletal proteins. (**A**) extracellular matrix proteins; (**B**) collagen type I, (**C**) cytoskeletal proteins.

The major portion of the identified differentially expressed proteins before and after decellularization belonged to cellular parts and organelles. Whereas around 20% of these differentially expressed proteins were in the extracellular space (Fig. 4). Among which, six ECM proteins were identified in the NPC-collagen microspheres, namely prolargin (PRELP); lactadherin (MFGE8); biglycan (BGN); fibromodulin (FMOD); hyaluronan and proteoglycan link protein 1 (HAPLN1); and secreted protein acidic and cysteine rich (SPARC). Five of these proteins (i.e., all apart from SPARC) were still maintained after decellularization (Fig. 5A). Moreover, PRELP, MFGE8 and BGN were maintained at high levels both before and after decellularization. The decellularization process alone did not disrupt the collagen type I matrix, whereas the collagen content slightly increased after decellularization (Fig. 5B). This might be because during the decellularization process, the detergent treatment partially cleaved bonds between the peptides, resulting in exposure of more amino and carboxylic groups and hence a higher hydroxyproline (HYP) content^17^. These results are consistent with our previous findings, when measured using the HYP assay^12^. In addition, although most of the cells were removed following decellularization, cellular components mainly cytoskeletal proteins still remained in the acellular matrix in small amounts (Fig. 5C).

### Effect of the NPC acellular matrix on hDFs

**Figure 6:**
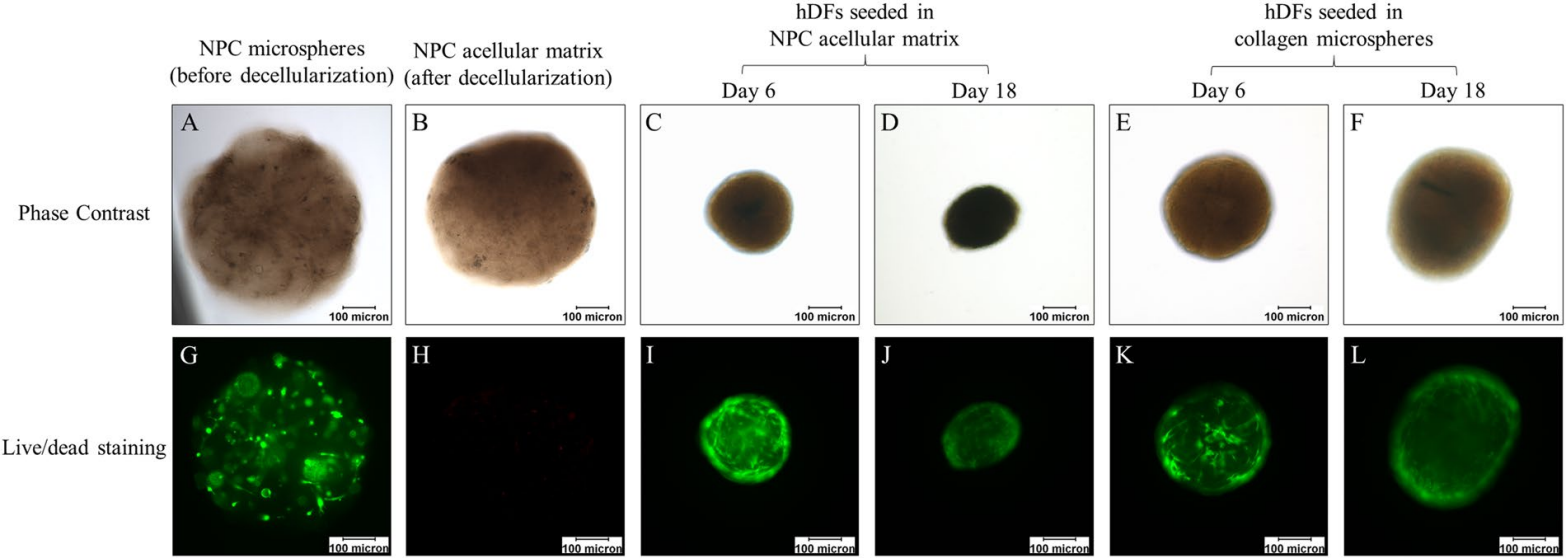
Viability of the NPC microspheres before and after decellularization, and of hDFs seeded in the NPC acellular matrix and collagen microspheres. (**A**–**F**) phase contrast images; (**G**–**L**) live/dead staining (green: live cells; red: dead cells); (**A** and **G**) NPC microspheres before decellularization; (**B** and **H**) NPC microspheres after decellularization; (**C,D** and **I,J**) hDFs seeded in NPC acellular matrix; (**E,F** and **K,L**) hDFs seeded in collagen microspheres; (**C,E,I** and **K**) Day 6; (**D,F,J** and **L**) Day 18. (Scale bars: 100 microns).

**Figure 7:**
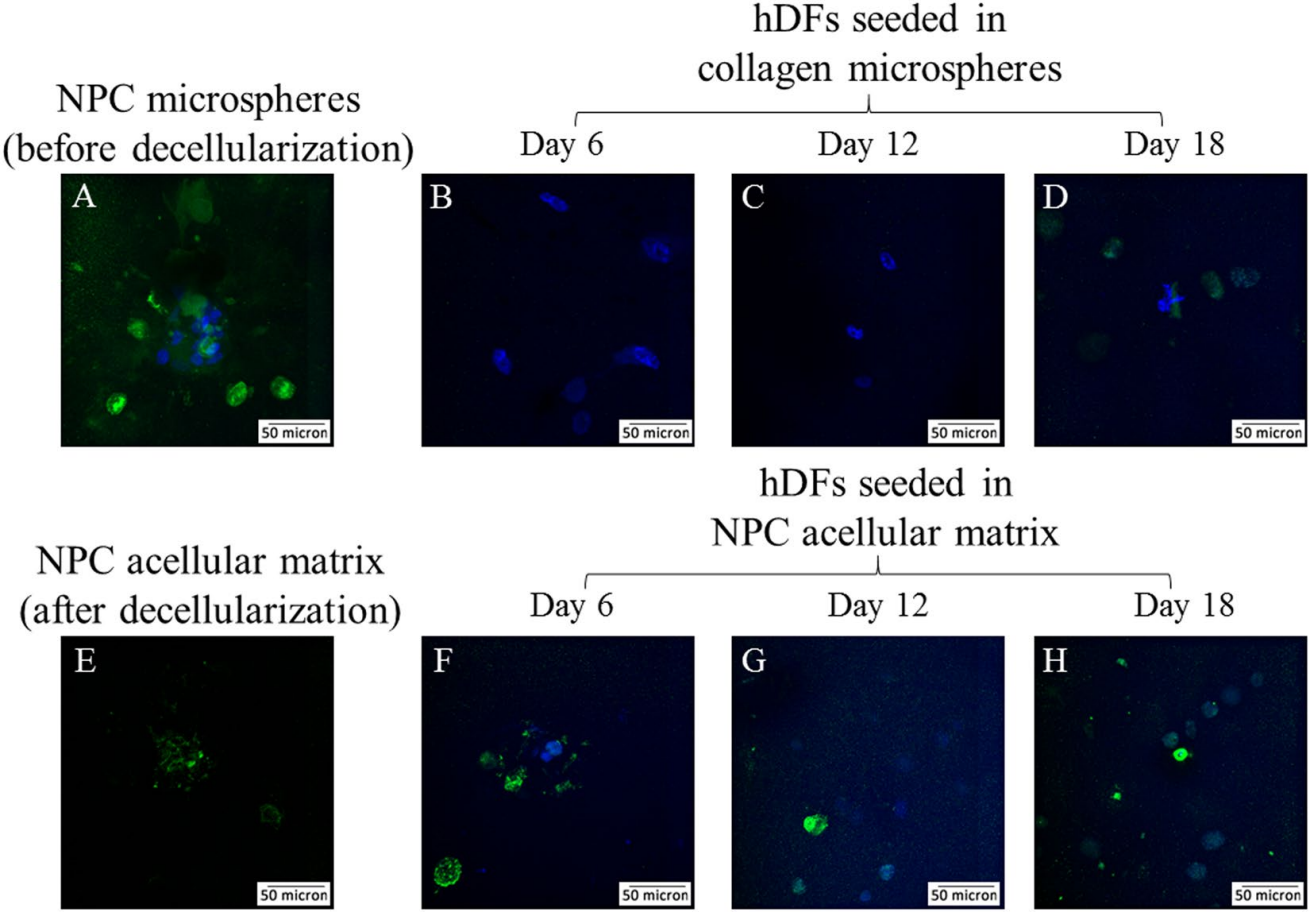
Immunofluorescent staining of keratin 19 in NPC microspheres before and after decellularization, and in hDFs seeded in NPC acellular matrix and collagen microspheres. (**A**) NPC microspheres before decellularization; (**B**–**D**) hDFs seeded in collagen microspheres; (**E**) NPC microspheres after decellularization; (**F**–**H**) hDFs seeded in NPC acellular matrix; (**B** and **F**) Day 6; (**C** and **G**) Day 12; (**D** and **H**) Day 18. (Green: keratin 19; blue: DAPI; scale bars: 50 microns).

**Figure 8:**
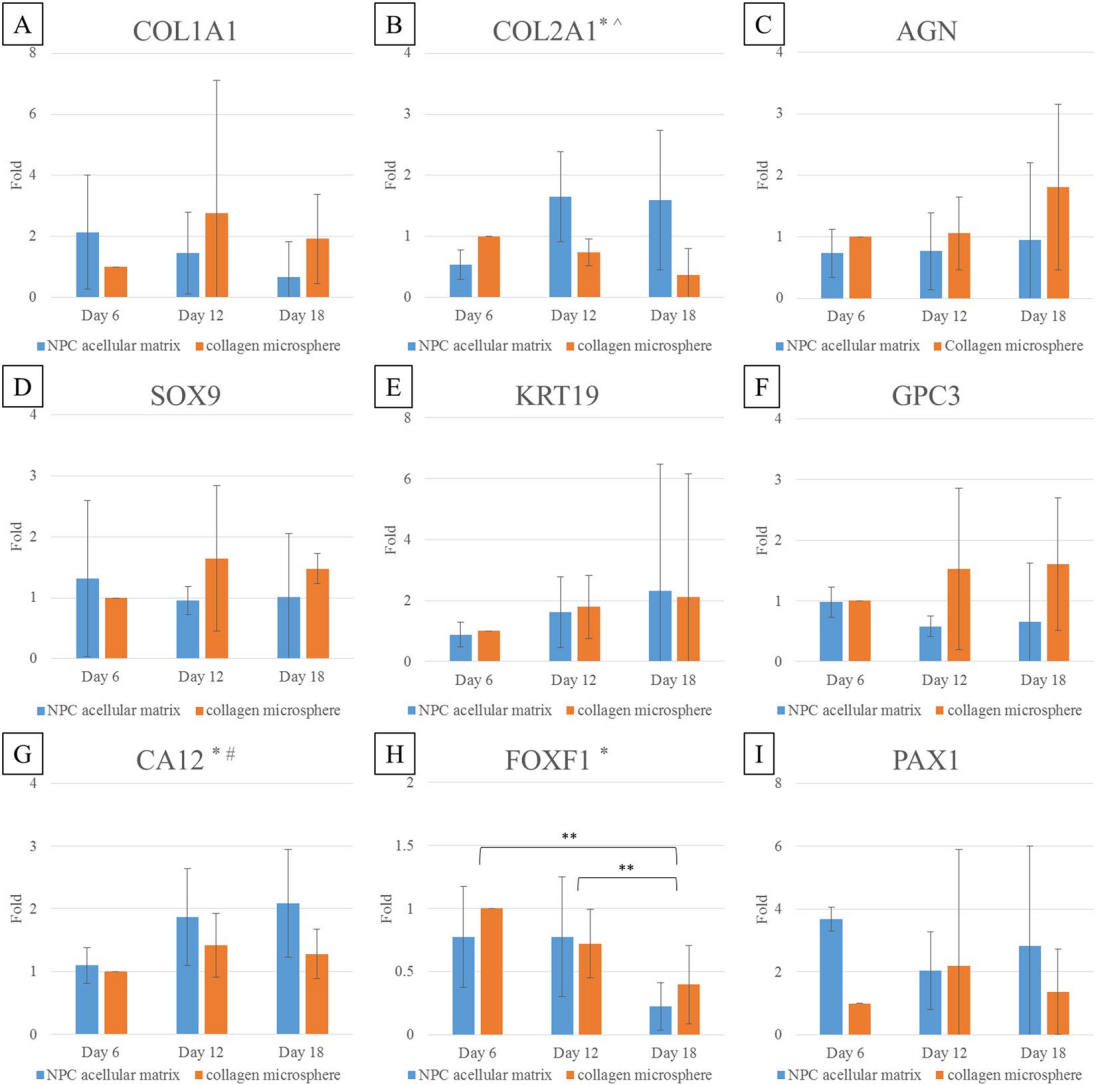
RNA expression of hDFs seeded in NPC acellular matrix and collagen microspheres evaluated by real-time RT-PCR. (**A**) collagen type I (COL1A1); (**B**) collagen type II (COL2A1); (**C**) aggrecan (AGN); (**D**) SOX9; (**E**) keratin 19 (KRT19); (**F**) glypican 3 (GPC3); (**G**) carbonic anhydrase XII (CA12); (**H**) forkhead box F1 (FOXF1); (**I**) Paired box 1 (PAX1). (All the data were normalized to the expression of genes in hDFs seeded in collagen microspheres at Day 6, and were expressed as mean ± standard deviation of n = 5 experiments. Two-way ANOVA showed significant difference (p < 0.05) in: *time, #group, ^interaction between time and group. **Bonferroni’s post hoc test showed significant different (p < 0.05).

As illustrated by live/dead staining, hDFs were able to penetrate into the centre of the decellularized NPC-collagen microspheres and survive for at least 18 days (Fig. 6). Keratin 19 (a potential marker for NPCs), showed positive staining both before and after decellularization (Fig. 7), which is consistent with the proteomic results (Fig. 5C). After the NPC acellular matrix was populated with hDFs, however, it was hard to determine if the keratin 19 observed, was from the remnant of the original NPC-derived matrix or the newly deposited of the seeded hDFs. This is because hDFs showed weak but positive staining for keratin 19 (Fig. 7D), while there was positive staining of keratin 19 after decellularization of NPC-derived matrix (Fig. 7E) and in hDFs seeded to the acellular NPC-derived matrix (Fig. 7F–H). As a result, gene expression rather than protein expression was used to evaluate the phenotypic markers in the newly seeded DFs (Fig. 8). hDFs exhibited a fibroblastic phenotype as they primarily produce collagen type I but not collagen type II^3^. However, when these cells were cultured in NPC-derived matrix, this expression pattern changed such that the expression of collagen type I decreased and the expression of collagen type II increased (Fig. 8A and B). Two-way ANOVA showed that both time (p = 0.025) and the interaction between time and group (p = 0.013) significantly affected the level of expression of the collagen type II gene. In addition, CA12, a potential phenotypic marker for NPCs^30^, was also significantly increased in the treatment group (Fig. 8G); two-way ANOVA showed that both time (p = 0.025) and group (p = 0.034) significantly affected the gene expression. However, the expression of another potential NPC marker, FOXF1, was significantly decreased over time (Fig. 8H). Indeed two-way ANOVA showed that the expression of FOXF1 was only significantly affected by the time factor (p = 0.002) but not the treatment groups (p = 0.338). Bonferroni’s post hoc test showed that the expression of FOXF1 at Day 6 and Day 12 was significantly different from that at Day 18 (at p = 0.002 and p = 0.023, respectively). In contrast, other chondrocytic or NPC phenotypic markers including aggrecan (Fig. 8C), SOX9 (Fig. 8D), keratin 19 (Fig. 8E), glypican 3 (Fig. 8F) and PAX1 (Fig. 8I) showed no statistically significant differences among different time points and treatment groups.

## Discussion

Collagen microencapsulation is a technique that produces self-assembled collagen–cell microspheres. Unlike a typical collagen gel or sponge, collagen microspheres serve as excellent cell delivery devices, which are stable, injectable and able to provide a protective, growth- and migration-supporting matrix for MSCs^18^. They have been used for culturing a wide range of cells, including embryonic stem cells^31^, chondrocytes^32^, human embryonic kidney 293 cells^33^ and rabbit NPCs^19^; and the cells can remodel the template collagen type I matrix. Thus, using a combination of microencapsulation and decellularization, a cell- or tissue-specific matrix niche can be reconstituted in a 3D configuration. When combined with a subsequent proteomics analysis, this can serve as a robust platform to study the complex matrix niche derived from a single population of cells including those from primary cultures, immortalized cell lines or following genetic manipulation. Therefore, this system is an especially useful *in vitro* model for studying cell niche factors including ECM, interactions between cells and ECM niche, and functional genomics, particularly on targets related to matrix deposition and remodelling. As a practical example, we used this platform to study the expression of genes in DFs after culturing and interacting with a complex NPC-derived matrix, with detailed composition analysis using proteomics.

Collagen and proteoglycans (PGs) are the major components of the ECM in the NP. As the template material of the microspheres also consisted of collagen type I, this was the most abundant protein identified, and it wasn’t affected by the decellularization process. Collagen type II was also identified in the NPC microspheres which might be from the NPCs directly (Supplementary Table S4). Large PG proteins, such as aggrecan, were not identified by mass spectrometry; however, the lack of peptides did not necessarily imply that a particular protein was absent from the sample^34^. Samples without deglycosylate before trypsin digestion may be one of the reasons for this. In addition, there were also many small PGs present in the NP. According to PANTHER, cytoskeletal and ECM proteins were the two major classes in the total protein. Six ECM proteins were identified in the NPC microspheres which were likely to be produced by NPCs: PRELP, MFGE8, BGN, FMOD, HAPLN1, and SPARC. All except SPARC were still maintained after decellularization; and PRELP, MFGE8 and BGN were the most abundant ones and were the least affected by the decellularization process. PRELP, BGN and FMOD have previously been shown to have distinct patterns of distribution in the disc^35^. They are members of the small leucine-rich repeat proteoglycan (SLRP) family, which play an important organisational role in the regulation of the IVD ECM during growth and maturation, and also during repair processes including fibrillogenesis, fibrosis and connective tissue remodelling^36^. They interact with specific regions on the surface of type I and II collagen fibrils to regulate matrix metalloproteinase (MMP) activity^37,38^. BGN is localised in pericellular regions^39^, and is considered to be a coordinator of matrix synthesis by proliferating cells; whereas FMOD is associated with collagen fibrils in the interstitial matrix^35^. HAPLN1 is known to interact with versican, which is a large ECM PG^40^. MFGE8 has not been widely studied in the IVD or cartilage, but it is reported to play a role in the clearance of apoptotic cells, as well as in the maintenance and repair of the epithelium in the intestine^41^; these processes might also occur in the IVD. Although most of the cells were removed by decellularization, some cellular components still remained in the acellular matrix. These included cytoskeletal proteins, which are known to contribute to mechanotransduction between cells and the ECM in the disc^42^.

In comparison with NPC-collagen microspheres, the amounts of some proteins appeared to increase after decellularization. This might be because cellular proteins in the NPC microspheres were highly abundant, making the detection of other less abundant proteins difficult (Supplementary Table S1). For example, in addition to collagen type I and II, a number of other types of collagen could be identified when the NPCs were released from the microspheres with collagenase (Supplementary Table S4). Aggrecan is a very abundant ECM component of the NP and as it is heavily glycosylated, this might interfere with the detection of other peptides by MS/MS. One solution might be to include a deglycosylation step (with a chaotropic reagent such as guanidine hydrochloride; GuHCl), prior to trypsin digestion^43^. The high level of collagen also made the identification of other less-abundant proteins difficult. In addition, almost all the peptides originating from collagen contained hydroxylated proline and/or lysine residues. As hydroxylation is a common posttranslational modification of this protein, providing sites for protein crosslinking, we included this in our search criteria. However, the posttranslational modification (and highly cross-linked network) of collagen made the proteomic analysis more complex. In order to minimize the influence of collagen as much as possible, we tried to digest the NPC-collagen microspheres and NPC-derived matrix with collagenase. As this enzyme cleaves at non-specific sites, only around 50% of the ECM proteins in the collagen matrix could be identified after treatment. However, following collagenase digestion, a lot of proteins expressed by NPCs could be identified after being released from the microspheres (Supplementary Table S1). This suggests that the large amounts of collagen were indeed suppressing the signals of other less abundant proteins.

It has been reported that DFs can exhibit different cell phenotypes when exposed to specific small-molecule activators^27^, growth factors^3,44,45^, or topographical structures^45,46^. This suggests that DFs might be able to alter their phenotypes into others when subjected to a suitable inducing agent or microenvironment. The NPC-derived matrix present a NP-like microenvironment, which might therefore provide the niches and signals required to affect the gene expression in hDFs. In this study, among the panel of potential human NPC marker genes selected, the expression of collagen type I decreased, suggesting a less fibroblastic phenotype; whereas the expression of a traditional NPC marker (i.e., collagen type II) and the non-chondrogenic NPC marker (i.e., CA12) significantly increased in hDFs re-seeded in the NPC-derived matrix, when compared with those in the collagen matrix control. This demonstrates that the NPC-derived matrix can alter the phenotype of hDFs, at least partially towards that of an NP-like lineage. Nevertheless, no significant differences in the expression of aggrecan, SOX9, keratin 19, glypican 3 or PAX1 genes was noted, and there was a decrease in the expression of FOXF1 when hDFs were cultured in the NPC-derived matrix for 18 days. The exact phenotype of NPCs is still largely unknown. Traditional markers for these cells, such as collagen type II, aggrecan and SOX9, are also used as key chondrogenic markers. In addition, although attempts have been made to find markers that can distinguish NPCs from articular chondrocytes (ACs), those that have been identified appear to be species-dependent^30,47–49^.For example, in the rat, keratin 19 and glypican 3 are specific for NPCs, and can therefore distinguish between NPCs and both annulus fibrosus cells (AFCs) and ACs^47^. However, in humans, only keratin 19 showed potential for identifying NPCs, but just in young or non-degenerated tissues^49^. In addition, on microarray analysis, there was only a ~2 fold difference in the expression of the keratin 19 gene in NPCs, when compared that in with ACs^30^. The bovine NPC marker, FOXF1^48^, as well as two additional novel human NPC markers, PAX1 and CA12, were all identified in the same study as having potential for use as NPC markers in humans^30^. PAX1 encodes a transcription factor that regulates pattern formation during embryogenesis in vertebrates^50^, whereas FOXF1 plays an important role in cell growth, proliferation, differentiation, and longevity^51–53^. Both of these genes are regulated by sonic hedgehog (SHH), which is expressed in the sclerotome during embryogenesis, and in the NP in the postnatal mouse^54,55^. The role of both these genes in the adult human IVD has yet to be examined. CA12 is a zinc-containing metalloenzyme, which catalyzes the reversible hydration of carbon dioxide and it plays a role in maintaining the intracellular ion and pH homeostasis^56^. Under hypoxic conditions, CA12 is regulated by hypoxia-inducible factor 1α (HIF-1α)^57^ and it probably helps to maintain homeostasis in the avascular, hypoxic, nutrient-deprived, and acidic environment of the adult human IVD^30^. To understand the molecular mechanisms for cell differentiation, further evaluations on factors that affect markers expression such as growth factors or inhibitors of SHH and HIF-1α, should be performed. On the other hand, to further characterize differentiated DFs, the cells could be released from the microspheres by collagenase and examine the expression profile of surface molecules including cell-cell and cell-matrix adhesion molecules by fluorescence-activated cell sorting (FACS) in comparison with that of NPCs and DFs. It was believed that adhesion molecules, including N-cadherin (mediates cell–cell interactions), integrin α5 and β1 (responsible for a signal switch from cell–cell to cell–matrix interactions in mature chondrocytes), play an important role in maintaining chondrogenic phenotype^58,59^. Analysis of these adhesion molecules in our 3D culture system should help us to know more about the interactions between cells and ECM.

In previous studies, we demonstrated that mature cells and committed MSCs could remodel the robust collagen meshwork in microspheres by simultaneously degrading the template collagen meshwork and depositing new ECM^32,60–62^. Here, we showed that upon removal of the encapsulated cells with an optimal decellularization method, the cell-specific matrix niche could be reconstituted in 3D configuration. This feature enables further investigation with regards to the composition of the cell-specific matrix niche using a proteomic approach because the starting material in our culture system was collagen type I; thus, any other ECM and biologic factors deposited on the matrix meshwork would be newly synthesized by the cells. This 3D microsphere-based culture model is easily scalable and can be used to screen for drugs for treating disc degeneration as well because the collagen meshwork has 400–500 nm mesh size, which is permissive to many small molecules, dyes, growth factors and even large proteins such as antibodies. The combined microencapsulation/decellularization platform can therefore be used to study the complex cell-derived matrix generated by a single population of cells. Such a physiologically relevant 3D culture model, although not exactly the same as that in the native tissue, presents an excellent model to study what NP cells, from healthy or degenerative discs, or NP-like cells deriving from stem or progenitor cells, are synthesizing or depositing, in response to other extrinsic factors, such as drugs, soluble factors, matrix factors and mechanical loading, or their interactions with other cell types, by systemically analyzing the protein deposited within the microspheres. Therefore, investigation of the differential responses of disc cells isolated from healthy/degenerative discs toward various risk factors for degeneration as well as potential candidates for treatments in such a 3D culture system would also be useful in pathoetiology and therapeutics of DDD information. In addition, it is a good *in vitro* model for studying functional genomics, particularly that related to matrix deposition and remodelling. Last but not the least, microspheres alone or the microspheres with the reconstituted acellular matrix niche can be served as carrier or scaffold for cell therapy, because of the size of them was able to control as injectable size (200–300 microns in diameter)^12^ and their solid appearance and reduced elastic modulus of around 10 kPa^61^, which is comparable to that of many soft connective tissues, including the nucleus pulposus. The strategy demonstrated here forms the basis of a robust model system for future investigations of the cell matrix niche, especially with regards to identifying the complex composition; the effects on altering the phenotype of cells exhibiting plasticity; the functions of genes related to matrix deposition and remodelling; and the optimization of cell-derived matrix as a scaffold for tissue engineering.

To conclude, here we demonstrated that the biomimetic ECM microenvironment of native NP tissue could be at least partially reconstituted and preserved via collagen microencapsulation and decellularization. In addition, we showed that the acellular matrix is a potentially valuable *in vitro* model for studying the cell matrix niche and its effect on altering the phenotype of cells (such as hDFs) that exhibit plasticity. We anticipate that this will contribute to the future development of high throughput microsphere or spheroid models for cell-matrix interaction studies and biomimetic scaffolds for tissue engineering applications.

## Methods

### Research Design

**Figure 9:**
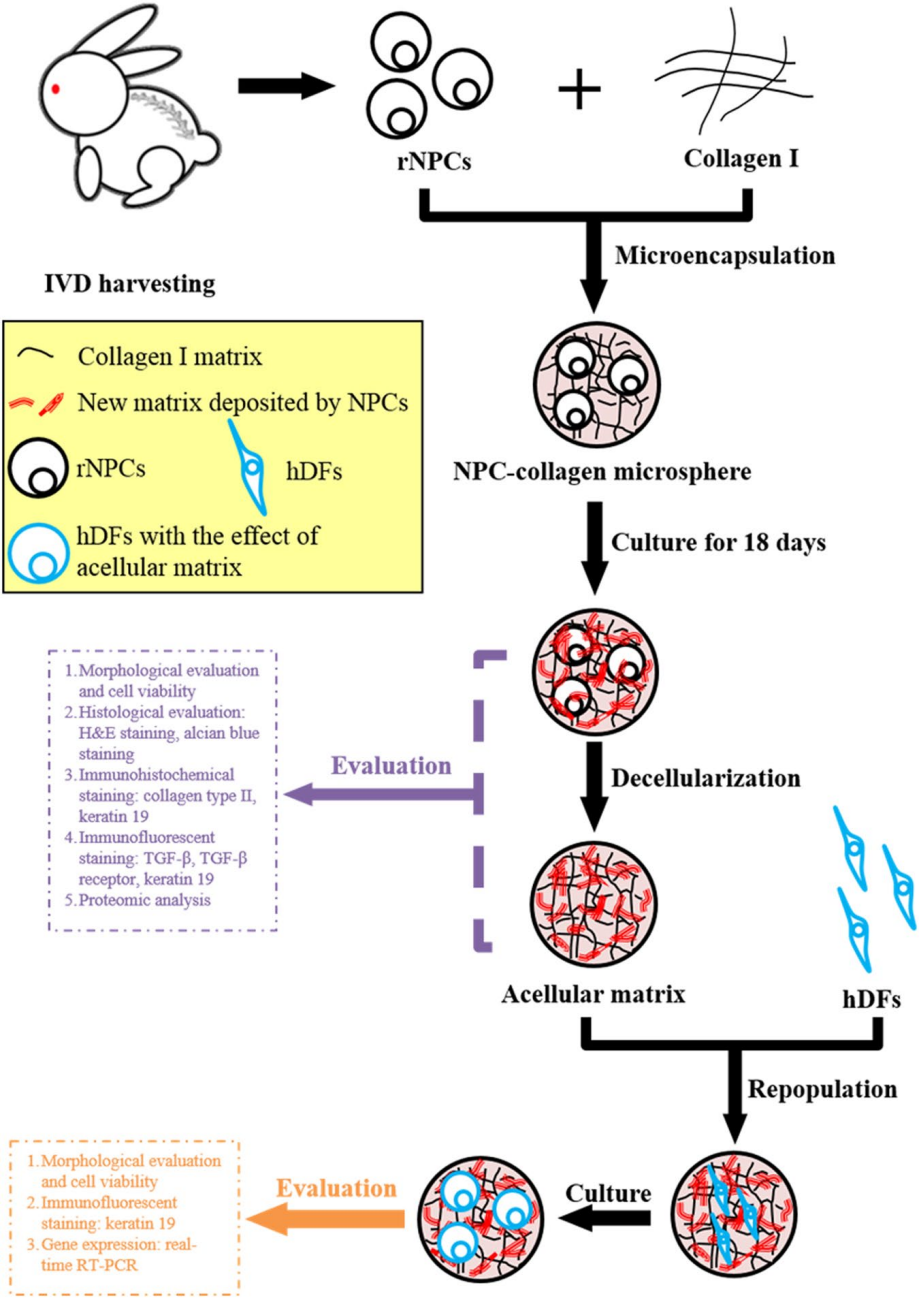
Schematic diagram on the overall design.

The overall research design of this study was shown in Fig. 9. NPCs were isolated from rabbit IVDs and expanded in monolayers before microencapsulating in collagen matrix to form NPC-collagen microspheres. After culturing for a period of time, NPC was removed by an established decellularization protocol whilst retaining most of the NPC-derived matrix. The NPC-derived matrix before and after decellularization was evaluated by morphological, histological and immunohistochemical methods. The composition of the NPC-derived matrix together with a number of control groups was studied by proteomic analysis. In order to investigate effect of the NPC-derived matrix on cellular phenotype changes, human DFs (hDFs) were repopulated on the acellular matrix and their phenotype was characterized by morphological evaluation, immunohistochemistry and real-time RT-PCR.

### Isolation and culture of NPCs

All the animal experiments were conducted with the approval of the Committee on the Use of Live Animals in Teaching and Research and all methods were performed in accordance with the relevant guidelines and regulations. IVD samples were harvested from T5 to L7 levels of New Zealand White rabbits aged 14–18 weeks (n = 8). The NPCs were isolated as previously described^12^. Briefly, the NP was separated from the other disc tissue and digested with 0.2% pronase (Sigma, St. Louis, MI, USA) for 1.5 hours, followed by treatment with 0.05% collagenase IA (Sigma) for another 16 hours. The digested NP was passed through a cell strainer (BD Biosciences, Franklin Lakes, NJ, USA) with a pore size of 40 μm and washed twice with phosphate-buffered saline (PBS). The isolated NPCs were seeded in culture dishes at 2.00 × 10^3^ cells/cm2 in full medium (Dulbecco’s modified Eagle’s medium with low glucose (DMEM-LG), supplemented with 10% fetal bovine serum (FBS; Gibco, Invitrogen, Carlsbad, CA, USA) and 1% 10,000-U penicillin/streptomycin (P/S; Gibco)), and incubated at 37 °C in an atmosphere of 5% CO_2_. The medium was changed every 2–3 days.

### Microencapsulation and 3D culture of NPCs

At 10 days post-isolation, the NPCs were trypsinized with 0.05% trypsin/ethylenediaminetetraacetic acid (EDTA; Gibco), after which they were microencapsulated in collagen microspheres at optimal culture conditions, including cell density and duration, as identified in a previous study^19^. In brief, rat-tail collagen type I (Becton Dickenson) was neutralized with 1 N sodium hydroxide (NaOH) and then adjusted to an optimal concentration of 2 mg/ml. The NPCs were then suspended in the neutralized collagen solution to produce cell-matrix mixtures with an initial cell density of 2.00 × 10^5^ cells/ml. Liquid droplets of 2.5 μl (containing ~500 cells/droplet) were dispensed onto the non-adhesive surface of ultraviolet-irradiated parafilm placed in Petri dishes. To induce the gelation of collagen, samples were incubated at 37 °C in a 5% CO_2_ atmosphere for 45 min. The gelated NPC-collagen microspheres were then suspended in DMEM-LG medium for 3D subculture (with regular medium replenishment), for 18 days to allow sufficient matrix remodelling before decellularization.

### Decellularization of NPC-collagen microspheres

The NPC-collagen microspheres were decellularized using a protocol optimized previously, involving a combination of the anionic detergent, Triton X-200, and the zwitterionic detergents, sulfobetaine-10 and -16 (i.e., SB-10 and SB-16; both from Sigma)^12^. In brief, the samples were washed with PBS and immersed in 10 mM phosphate-buffered 50 mM sodium solution containing 50 mM SB-10 for 30 min. After rinsing with 50 mM phosphate-buffered 100 mM sodium solution, the samples were then immersed in 10 mM phosphate-buffered 50 mM sodium solution containing 0.6 mM SB-16 and 0.14% Triton X-200 for 30 min. After washing with PBS three times, the above steps were repeated, but the incubation times with detergent were reduced to 15 min. All the steps were carried out with gentle shaking at room temperature.

### Mass spectrometry analysis of the NPC-derived matrix

To evaluate the chemical components of the NPC-derived matrix, NPC-collagen microspheres were decellularized at Day 18 and samples both before and after decellularization were collected for mass spectrometry analysis. Collagen microspheres without NPCs were used as a negative control, and 150 microspheres per condition were used. The samples were dissolved in the lysis buffer and heated at 100 °C for 10 min to denature the proteins. The total protein concentration was determined by the bicinchoninic acid assay (Pierce^TM^ BCA Protein Assay Kit, Thermo Fisher Scientific, Bremen, Germany). The samples (30 µg) were loaded into sodium dodecyl sulfate-polyacrylamide gel electrophoresis (SDS-PAGE). Each gel lane containing the separated sample was cut into 4 similar-sized pieces. Each piece was washed with 25 mM ammonium bicarbonate buffer and then treated with dehydration solution consisting of a mixture of acetonitrile and 50 mM ammonium bicarbonate (at a 2:1 ratio), to remove the Coomassie blue on the protein. The resulting pieces of gel were treated with 10 mM dithiothreitol for 45 min at 56 °C, and then with 55 mM iodacetamide for 30 min in the dark, to reduce the disulphide bonds on the cysteines and to alkylate the reduced cysteines, respectively. The pieces of gel were then incubated with dehydration solution again before being incubated with 20 ng/μl of sequencing grade modified trypsin (Promega Corporation, Madison, Wisconsin, USA) in 25 mM ammonium bicarbonate buffer (pH 7.8) overnight at 37 °C. The resulting solutions contained mixtures of tryptic peptides were collected. The remaining peptides were further extracted from the gel pieces by treatment with the dehydration solution and the extracted solutions were collected and combined with that previously collected. Liquid chromatography tandem mass spectrometry (LC-MS/MS) analysis with a full scan spectrum from 400 m/z to 2000 m/z was performed using the LTQ Velos mass spectrometer (Thermo Fisher Scientific) coupled with a Thermo Accela LC for each fraction. The OMSSA (open mass spectrometry search algorithm) search engine was used to perform database searches against a combined protein database including the RAT and RABBIT reviewed protein databases from UniProt. Two searches were performed: one with proline hydroxylation as a variable modification against only collagen sequences, and one without such a modification against all sequences. The search results from all four fractions and the LC/MS technical replicates were combined and further processed with the Trans-Proteomic Pipeline (TPP) software suite, comprising PeptideProphet, iProphet and ProteinProphet. The protein false discovery rate (FDR) cut-off was set at 0.01. Since the rabbit proteome is not well characterized, the functions of the uncharacterized rabbit proteins were predicted by performing “The Basic Local Alignment Search Tool” (BLAST) against the “Mammals” sequences in the UniProt database (http://www.uniprot.org/blast/) to find similar sequences in other mammals for which the function is known.

For label-free quantitative proteomics analysis by spectral counting, MS/MS spectral counts for identified proteins were retrieved from search results. Quantitative proteomics were performed in two groups: (1) NPC sample of acellular matrix compared with control sample of collagen bead after decellularization; (2) NPC sample of NPC-collagen microspheres compared with control sample of collagen bead. The spectral count of each protein was normalized by the spectral count of trypsin which was assumed to be present at the same concentration in all samples. Proteins with spectral counts ≤10 were removed.

### Data annotation

Gene Ontology (GO) analysis was performed to identify the protein classes and cellular components associated with the identified proteins using the PANTHER (Protein ANalysis THrough Evolutionary Relationships) classification system (www.pantherdb.org)^63–65^.

### Repopulation of hDFs on the NPC-derived matrix

In order to test the effect of NPC-derived matrix niche, a cell with plasticity was used to evaluate the phenotypes. To distinguish the cell-derived matrix and newly made matrix, cells from a different species was used. Normal human dermal fibroblasts (hDFs) from Clonetics^TM^ Dermal Fibroblast Cell Systems (Lonza Walkersville, Inc., USA) were used in this study. hDFs were cultured in Dulbecco’s modified Eagle’s medium with high glucose (DMEM-HG), supplemented with 10% FBS and 1% 10,000-U P/S, and incubated at 37 °C in an atmosphere comprising 5% CO_2_, with regular replenishment of medium every 2–3 days. Upon reaching 80–90% confluence, the cells were detached by treatment with 0.05% Trypsin/EDTA and sub-cultured. Cells at P4 were used for subsequent collagen encapsulation or recellularization of the decellularized NPC-collagen microspheres.

To evaluate the efficacy of the NPC-derived matrix, the NPC-collagen microspheres were decellularized as described above. They were then equilibrated with full medium of DFs for 1 hour, after which they were transferred to a parafilm-coated Petri dish without medium, and 0.5 μl of hDF solution (at a concentration of 2 × 10^6^ cell/ml) was applied to each microsphere. The hDFs were allowed to adhere on the microspheres for 45 min, after which they were supplied with DMEM-HG medium. As a control, hDFs at an initial cell density of 500 cells/2.5 μl droplet were encapsulated in 2 mg/ml collagen alone.

### Morphological evaluation and cell viability

The morphology of the NPC-collagen microspheres before and after decellularization, the NPC-derived ECM following repopulation with hDFs, and the hDF-collagen microspheres were observed via a Leica phase-contrast light microscope (Leica DMIL, Solms, Germany) and images were captured with a Nikon COOLPIX5400 camera (Nikon, Tokyo, Japan). The viability of the NPCs in the microspheres before and after decellularization, the hDFs seeded in the NPC-derived decellularized matrix and the hDFs seeded in the collagen microspheres was determined by live/dead staining at 6 and 18 days. Cells were incubated for 45 min in the dark with 2 μM calcein acetoxymethylester and 4 μM ethidium homodimer-1 (LIVE/DEAD Viability/Cytotoxicity Kit; Molecular Probes, Invitrogen, Carlsbad, CA, USA), to stain the live and dead cells, respectively. The microspheres were then washed with PBS and examined under a Nikon inverted fluorescent microscope Eclipse TE2000-U equipped with a SPOT FLEX camera (Diagnostic Instruments, Sterling Heights, MI, US).

### Histological, histochemical, immunohistochemical and immunofluorescent evaluation

To characterize the NPC-derived matrix, the NPC-collagen microspheres before and after decellularization, were analysed by histological, histochemical, immunohistochemical (IHC) and immunofluorescent (IF) evaluation. First, the samples were washed three times with PBS, fixed with 4% PBS-buffered paraformaldehyde (PFA; BDH, Radnor, PA, USA) for 1 hour and embedded in paraffin. Sections of 5 μm thickness were then produced using a microtome (Microm, Walldorf, Germany). Routine hematoxylin and eosin (H&E) staining was used for revealing the cell morphology, while alcian blue (in combination with nuclear fast red as a counterstain) was used to show the GAG-rich region in the microspheres.

In the IHC and IF experiments, antigen retrieval was first required to expose the antigenic sites. For IHC, the sections were incubated with 0.5% pepsin (Sigma) at 37 °C for 30 min, whereas for IF, the sections were incubated in citrate buffer (10 mM sodium citrate, 0.05% Tween 20, pH 6.0) at 95 °C for 20 min.

The expression of the NPC markers, collagen type II and keratin 19, was evaluated by IHC staining. The sections were treated with methanol containing 3% hydrogen peroxide for 30 min in the dark to block endogenous peroxidase activity, after which any nonspecific background staining was blocked with 2% normal horse serum (Vector Laboratories, Burlingame, CA, USA) for 30 min at room temperature. The sections were then incubated at 4 °C overnight with mouse monoclonal antibodies against collagen type II (Calbiochem, Merck, Germany) or keratin 19 (Progen Biotechnik GmbH, Heidelberg, Germany) at dilutions of 1:1000 and 1:10, respectively, in PBS containing 2% bovine serum albumin (BSA; Sigma). The sections were washed with PBS and then incubated with biotinylated secondary antibody anti-mouse IgG (Dako, Glostrup, Denmark), at dilutions of 1:400 and 1:500 for the anti-collagen type II and anti-keratin 19 primary antibodies, respectively, for 30 min at room temperature. Positive labelling was visualized with the Vectastain ABC kit (Vector Laboratories), after which the samples were counterstained with hematoxylin (Vector Laboratories) and then mounted under coverslips with DePex (Advanced technology & industrial Co. Ltd, Hong Kong, China).

The presence of TGF-β and TGF-β receptor I was evaluated by IF staining. Following antigen retrieval, the sections were incubated with 5% BSA for 1 h at room temperature. They were then incubated with a mouse monoclonal antibody against TGF-β1, 2, 3 (R&D systems, MN, USA), or a goat polyclonal antibody against TGF-β receptor I (Abcam) at dilutions of 1:60 and 1:500, respectively, in 5% BSA at 4 °C overnight. After washing, the sections were then incubated for 1 h at room temperature with Alexa Fluor® 546-tagged goat anti-mouse IgG or Alexa Fluor® 488-tagged donkey anti-goat IgG (Molecular Probes, Invitrogen, Carlsbad, CA, USA) at dilutions of 1:200 and 1:400, respectively, in 5% BSA. The sections were then mounted under coverslips with Fluoro-Gel II mounting medium (Electron Microscopy Sciences, PA, USA) containing 4, 6-diamino-2-phenylindole (DAPI).

To characterize the effect of hDFs on the NPC-derived matrix, hDF-seeded acellular NPC-collagen microspheres and hDF-collagen microspheres were fixed at 6, 12 and 18 days with PFA for 1 hour after which the expression of keratin 19 was evaluated by IF staining. Intact microspheres were washed with PBS containing 0.05% Tween 20 (PBST) three times after which any nonspecific background staining was blocked with PBS containing 5% BSA for 1 h at room temperature. The microspheres were then incubated with the mouse anti-keratin 19 described above (at a 1:10 dilution), at 4 °C overnight, followed by Alexa Fluor® 488-tagged donkey anti-mouse IgG (at a 1:400 dilution) for 1 h at room temperature. Both antibodies were prepared in PBS containing 5% BSA. The microspheres were mounted to glass bottom culture dishes (MatTek Corporation, MA, US) under Fluoro-Gel II mounting medium containing DAPI. Following IF, representative images from the sections and intact microspheres were captured either by confocal fluorescence microscopy using an LSM 710 (Carl Zeiss, Germany), or by Nikon inverted fluorescent microscope Eclipse TE2000-U using a SPOT FLEX digital camera.

### Expression of phenotypic NPC marker genes by real-time RT-PCR

To evaluate the effect of the NPC acellular matrix on alternation of phenotype of hDFs, real-time RT-PCR was performed on the decellularized NPC-collagen microspheres at 6, 12 and 18 days after they were seeded with hDFs. The expression levels of the following nine genes were chosen to evaluate if the hDFs had exhibited any phenotypic changes: collagen type I and II (COL1A1 and COL2A1); aggrecan (AGN); SOX-9; keratin 19 (KRT19); glypican 3 (GPC3); carbonic anhydrase XII (CA12); forkhead box F1 (FOXF1); and paired box 1 (PAX1). COL1A1 is a specific marker for fibroblasts, COL2A1, AGN and SOX-9 are traditional chondrocytic markers for NPCs while KRT19, GPC3, CA12, FOXF1 and PAX1 are all “potential” specific markers for NPCs^30,47^. Glyceraldehyde-3-phosphate dehydrogenase (GAPDH; Applied Biosystems) was used as the endogenous control (Supplementary Table S5). RNA was extracted from 100 microspheres per condition. Reverse transcription of total RNA to cDNA was conducted with a TaqMan Reverse Transcription kit (Applied Biosystems, Foster City, CA). For COL1A1, SOX-9, KRT19 and GPC3, real-time PCR was run with the TaqMan® Gene Expression Master Mix (Applied Biosystems) using standard thermal conditions (Applied Biosystems). For COL2A1, AGN, CA12, FOXF1 and PAX1, real-time PCR was run with the Power SYBR® Green PCR Master Mix (Applied Biosystems). Data analysis was conducted using the relative quantification method, where C_t_ values from hDFs cultured on microspheres at Day 6 were used as the experimental calibrator. Day 0 was not used because the values obtained at this time-point were barely detectable.

### Statistical analysis

Gene expression levels were reported as mean ± standard deviation. Using SPSS 22 (SPSS Inc., Chicago, IL, USA), two-way analysis of variance (ANOVA) with the appropriate post hoc test was used to reveal differences in the levels of gene expression among the various treatment groups and the significance level was set at 0.05.

### Data availability statement

All data generated or analysed during this study are included in this published article and its Supplementary Information files.

## Electronic supplementary material

Supplementary Information
