# Supplementary material for: Proteomic Analysis of Nucleus Pulposus Cell-derived Extracellular Matrix Niche and Its Effect on Phenotypic Alteration of Dermal Fibroblasts
Source: Sci Rep. 2018 Jan 24;8:1512. doi: 10.1038/s41598-018-19931-9 (PMC5784136; doi:10.1038/s41598-018-19931-9)
Supplement: Supplementary file 1 — Supplementary Information [file 41598_2018_19931_MOESM1_ESM.pdf]

Supplementary Information

Proteomic Analysis of Nucleus Pulposus Cell-derived Extracellular Matrix Niche and Its Effect on Phenotypic Alteration of Dermal Fibroblasts

Minting Yuan [1], Pei-Jing Pai [2], Xiaofen Liu [3], Henry Lam [2,3,*], Barbara P. Chan [1,*]

[1] Tissue Engineering Lab, Department of Mechanical Engineering, The University of Hong Kong, Pokfulam Road, HKSAR China

[2] Department of Chemical and Biomolecular Engineering, The Hong Kong University of Science and Technology, Clear Water Bay, HKSAR China

[3] Division of Biomedical Engineering, The Hong Kong University of Science and Technology, Clear Water Bay, HKSAR China

[*] Corresponding email: bpchan@hku.hk & kehlam@ust.hk

This file includes Supplementary Table S1-S5.

List of supplementary tables

Supplementary Table S1 Protein ID summary for NPC samples and controls
Supplementary Table S2 Information about the proteins with spectral counts > 10
Supplementary Table S3 Proteins in the negative control group (i.e., collagen beads)
Supplementary Table S4 The types of collagen identified in NPC that were released from NPC-collagen microspheres with collagenase
Supplementary Table S5 Primers used for RT-PCR analysis

Supplementary Table S1 Protein ID summary for the NPC samples and controls

	Sample name	Proteins identified at FDR 0.01
NPC samples	Acellular matrix 1	120
	Acellular matrix 2	146
	NPC-collagen microspheres	220
	Acellular matrix treated with collagenase 1	78
	Acellular matrix treated with collagenase 2	63
	NPC-collagen microspheres treated with collagenase 1	749
	NPC-collagen microspheres treated with collagenase 2	382
Controls	Collagen bead after decellularization	80
	Collagen bead	120
	Collagen bead treated with collagenase	68

Supplementary Table S2 Information about the proteins with spectral counts > 10

Gene Name	Protein Name	Spectral counts (normalized)		Spectral counts (normalized)		PANTHER protein class
		NPC microspheres	Collagen bead	NPC acellular matrix	Collagen bead after decellularization	
COL1A2	Collagen alpha-2(I) chain	4406	5345	5175	5421	
COL1A1	Collagen alpha-1(I) chain	4348	6714	5980	7761	
COL2A1	Collagen alpha-1(II) chain	107	132	0	0	
VIM	Vimentin	619	50	664	52	2, 16
TOM1L2	Target of myb1-like protein 2 (TOM1-like protein 2)	241	191	81	195	3, 13
FARP2	FERM, RhoGEF and pleckstrin domain protein 2	239	0	0	0	9
PRELP	Prolargin	133	0	164	0	1, 18
MFGE8	Lactadherin (Milk fat globule-EGF factor 8 protein)	83	0	87	0	1, 3, 4, 6, 8, 9, 11, 12, 18
P4HB	Protein disulfide-isomerase (beta-subunit of prolyl 4-hydroxylase)	75	0	17	0	
PDIA3	Protein disulfide-isomerase A3	73	0	0	0	
PDIA6	Protein disulfide-isomerase A6	24	0	0	0	
ANXA1	Annexin A1	19	0	0	0	
ANXA2	Annexin A2	0	0	29	10	
ANXA4	Annexin A4	23	0	0	0	
ANXA5	Annexin A5	36	0	0	0	
ANXA8	Annexin A8	74	0	8	0	
BGN	Biglycan	63	0	131	0	1, 8, 18
MYH1	Myosin-1	0	18	8	26	2, 9, 15
MYH8	Myosin-8	0	0	8	0	2, 9, 15
MYH9	Myosin-9	49	0	128	16	2, 9, 15
FMOD	Fibromodulin (Fragment)	43	0	19	0	1, 18
CALR	Calreticulin	37	0	0	0	10
ACTB	Actin, cytoplasmic 1	36	46	28	30	2
ACTA2	Actin, aortic smooth muscle	15	0	13	15	2
CKAP4	Cytoskeleton-associated protein 4	32	0	61	0	
TPM1	Tropomyosin 1, alpha	5	12	9	0	2
TPM2	Tropomyosin beta chain	29	0	8	0	2
LOC100349893 (TPM3)	Tropomyosin alpha-3 chain	5	20	0	0	2
TPM4	Tropomyosin alpha-4 chain	2	18	4	13	2
CTSB	Cathepsin B	18	0	0	0	11
CTSD	Cathepsin D	27	0	0	0	11
ACTN4	Alpha-actinin-4	23	5	0	0	2
HIST1H2AK	Histone H2A	22	1	22	0	7
HIST1H2BO	Histone H2B	23	0	0	0	7
HIST1H4C	Histone H4 (Fragment)	0	0	26	0	
HAPLN1	Hyaluronan and proteoglycan link protein 1	21	0	32	0	1
LOC100360413	Elongation factor 1-alpha	20	0	0	0	7, 9, 11
EEF1D	Elongation factor 1-delta	0	0	6	0	7
HSPA5	Heat Shock 70kDa Protein 5 (78 kDa glucose-regulated protein)	19	0	17	0	
HSPD1	60 kDa heat shock protein, mitochondrial	17	0	0	0	14
HSP90AA1	Heat shock protein HSP 90-alpha	0	0	10	0	14
HSP90B1	Endoplasmin (Heat shock protein 90kDa beta member 1)	0	0	12	0	14
KRT1	Keratin, type II cytoskeletal 1	14	26	19	22	2, 16
KRT6A	Keratin, type II cytoskeletal 6A	19	19	21	22	2, 16
KRT8	Keratin, type II cytoskeletal 8 (Fragment)	18	0	12	0	2, 16
KRT18	Keratin, type I cytoskeletal 18 (Fragment)	13	0	15	11	2, 16
KRT19	Keratin, type I cytoskeletal 19	0	0	15	0	2, 16
FN1	Fibronectin	33	22	24	0	8
YWHAE	14-3-3 protein epsilon	17	22	3	12	14
SFN	14-3-3 protein sigma	0	0	9	0	14
YWHAZ	14-3-3 protein zeta/delta	14	7	0	0	14
TUBB2A	Tubulin beta-2A chain	17	0	10	0	2
TUBB4B	RCG45400 (Predicted: tubulin beta-4B chain)	15	0	0	0	2
ENO1	Enolase (phosphopyruvate hydratase/ 2-phosphoglycerate dehydratase)	17	0	8	2	5
SPARC	Secreted protein acidic and cysteine rich (SPARC)	15	0	0	0	1, 6, 8
HTRA1	Serine protease HTRA1	15	0	20	0	11, 14
RCN3	Reticulocalbin-3 (Fragment)	15	0	0	0	10
TPI1	Triosephosphate isomerase	14	0	0	0	
PKM	Pyruvate kinase	14	0	0	14	
CRYAB	Alpha-crystallin B chain	14	0	12	0	14
LOC100362298	Protein LOC100362298 (Predicted: ribosomal protein S18-like)	13	0	0	0	7
SERPINH1	Serpin H1	12	0	0	0	9
CCDC130	Coiled-coil domain-containing protein 130	12	21	11	16	7
ERP29	Endoplasmic reticulum resident protein 29 (Fragment)	12	0	0	0	13
RPS19	40S ribosomal protein S19	12	0	0	0	7
ALB	Serum Albumin	6	32	0	42	12
LMNA	Lamin A	0	0	43	0	2, 16
UBB	Polyubiquitin-B	0	0	6	0	
JUP	Junction plakoglobin	0	0	16	0	2, 6, 8, 17
PLEC	Plectin	0	0	18	0	2
F2	Prothrombin; Coagulation factor II	0	0	1	75	11
PKP1	Plakophilin-1	0	0	19	0	2
DSP	Desmoplakin	0	0	26	0	2
LOC100349551	Glyceraldehyde-3-phosphate dehydrogenase (GAPDH)	0	0	5	13	4

Supplementary Table S3 Proteins in the negative control group (i.e., collagen beads)

Protein IDs with spectral counts of negative control (collagen bead) two times greater than those in the experimental groups were regarded as being impurities or contaminants (marked with underline).

Gene Name	Protein Name	Spectral counts (normalized)		Spectral counts (normalized)	
		NPC microspheres	Collagen bead	NPC acellular matrix	Collagen bead after decellularization
COL1A2	Collagen alpha-2(I) chain	4406	5345	5175	5421
COL1A1	Collagen alpha-1(I) chain	4348	6714	5980	7761
COL2A1	Collagen alpha-1(II) chain	107	132	0	0
VIM	Vimentin	619	50	664	52
TOM1L2	Target of myb1-like protein 2 (TOM1-like protein 2)	241	191	81	195
ANXA2	Annexin A2	0	0	29	10
MYH1	Myosin-1	0	18	8	26
MYH9	Myosin-9	49	0	128	16
ACTB	Actin, cytoplasmic 1	36	46	28	30
ACTA2	Actin, aortic smooth muscle	15	0	13	15
TPM1	Tropomyosin 1, alpha	5	12	9	0
LOC100349893 ; (TPM3)	Tropomyosin alpha-3 chain	5	20	0	0
TPM4	Tropomyosin alpha-4 chain	2	18	4	13
ACTN4	Alpha-actinin-4	23	5	0	0
HIST1H2AK	Histone H2A	22	1	22	0
KRT1	Keratin, type II cytoskeletal 1	14	26	19	22
KRT6A	Keratin, type II cytoskeletal 6A	19	19	21	22
KRT18	Keratin, type I cytoskeletal 18 (Fragment)	13	0	15	11
FN1	Fibronectin (Fragment)	19	11	24	0
FN1	Fibronectin	14	11	0	0
YWHAE	14-3-3 protein epsilon	17	22	3	12
YWHAZ	14-3-3 protein zeta/delta	14	7	0	0
ENO1	Enolase (phosphopyruvate hydratase/ 2-phosphoglycerate dehydratase)	17	0	8	2
PKM	Pyruvate kinase	14	0	0	14
CCDC130	Coiled-coil domain-containing protein 130	12	21	11	16
ALB	Serum Albumin	6	32	0	42
F2	Prothrombin; Coagulation factor II	0	0	1	75
LOC100349551	Glyceraldehyde-3-phosphate dehydrogenase (GAPDH)	0	0	5	13

Supplementary Table S4 The types of collagen identified in NPCs that were released from NPC-collagen microspheres with collagenase

Gene Name	Protein Name	Spectral counts (normalized)
COL1A1	Collagen alpha-1(I) chain	1080
COL1A2	Collagen alpha-2(I) chain	498
COL12A1	Collagen alpha-1(XII) chain	109
COL2A1	Collagen alpha-1(II) chain	38
COL8A1	Procollagen, type VIII, alpha 1 (Predicted)	13
COL11A1	Collagen alpha-1(XI) chain	8

Supplementary Table S5 Primers used for RT-PCR analysis

Reagent: Power SYBR® Green PCR Master Mix

Gene (Abbr.)	Forward Primer	Reverse Primer
Collagen type II (COL2A1)	TTTCCCAGGTCAAGATGGTC	CTGCAGCACCTGTCTCACCA
Aggrecan (AGN)	ACAGCTGGGGACATTAGTGG	GTGGAATGCAGAGGTGGTTT
Carbonic anhydrase XII (CA12)	CGTGCTCCTGCTGGTGATCT	AGTCCACTTGGAACCGTTCACT
Forkhead box F1 (FOXF1)	AAGCCGCCCTATTCCTACATC	GCGCTTGGTGGGTGAACT
Paired box 1 (PAX1)	TGGCCCTCGGCACACTC	GCCCCTGTTTGCTCCATAAA
GAPDH	GAGTCAAGGGATTTGGTCGT	TTGATTTTGGAGGGATCTCG

Reagent: TaqMan® Gene Expression Master Mix

Gene (Abbr.)	Assay ID of Applied Biosystem
Collagen type I (COL1A1)	Hs00164004_m1
SOX-9	Hs00165814_m1
Keratin 19 (KRT19)	Hs00761767_s1
Glypican (GPC3)	Hs00170471_m1
GAPDH	Hs99999951_M1
